# The variation in the major constituents of the dried rhizome of *Ligusticum chuanxiong* (*Chuanxiong*) after herbal processing

**DOI:** 10.1186/s13020-016-0098-5

**Published:** 2016-05-24

**Authors:** Tao Yi, Jia-Yan Fang, Lin Zhu, Yi-Na Tang, Hong Ji, Ya-Zhou Zhang, Ju-Cheng Yu, Xiao-Jun Zhang, Zhi-Ling Yu, Zhong-Zhen Zhao, Hu-Biao Chen

**Affiliations:** School of Chinese Medicine, Hong Kong Baptist University, Hong Kong Special Administrative Region, Hong Kong, People’s Republic of China; School of Pharmaceutical Science, Guangzhou Medical University, Guangzhou, People’s Republic of China; Guizhou Institute of Technology, Guiyang, People’s Republic of China; School of Chinese Medicine, Guangzhou University of Traditional Chinese Medicine, Guangzhou, People’s Republic of China

## Abstract

**Background:**

Rhizoma Chuanxiong (RC; *Chuanxiong*), which is the dried rhizome of *Ligusticum chuanxiong* (Umbelliferae), is commonly used in Chinese medicine (CM) for improving blood circulation and dispersing blood stasis. RC is usually processed before use in clinical practice to enhance its therapeutic efficacy. This study aimed to investigate the temporal variations of the major constituents of RC by HPLC-DAD-MS during herbal processing to investigate the effects of an adjuvant (e.g., wine), steaming vs stir-frying and the optimal processing time.

**Methods:**

An HPLC-DAD-MS method was developed to determine the major constituents of the RC processed by one of the four processing methods, i.e., stir-frying, steaming, stir-frying with rice wine and steaming with rice wine. Processing was conducted over 60 min. Six major compounds, namely ferulic acid, senkyunolide I, senkyunolide H, senkyunolide A, Z-ligustilide and levistolide A, were selected as markers to analyze the effects on the markers’ levels of the different processing methods and optimize the processing time.

**Results:**

The results indicated that (a) processing with wine had no discernible impact on the amounts of the six chemical markers in RC; (b) the amounts of the major constituents of RC subjected to steam processing were higher than those of the RC subjected to stir-fry processing.

**Conclusion:**

Among the four different methods evaluated for RC processing, steaming was better and the optimal time for steaming RC was 40 min.

## Background

Herbs can be processed in a variety of different ways, including parching, stir-baking with liquid, calcination, roasting in fresh cinders, steaming and boiling. Certain processing methods can affect the properties and functions of herbs by reducing their side effects and enhancing their biological efficacy, and can therefore be used to facilitate herbal decoction preparation and preservation. Raw Chinese medicinal herbs and teas are subjected to extensive processing procedures prior to being used in clinical prescriptions or for the preparation of proprietary Chinese medicines [1, 2]. Assessing the effects of various processing methods and optimizing the processing parameters are important to Chinese medicine (CM) for producing the most effective herbs from raw herbal materials.

Rhizoma Chuanxiong (RC; *Chuanxiong*), which is the dried rhizome of *Ligusticum chuanxiong* Hort (Umbelliferae), is used in CM for the treatment of cerebrovascular and cardiovascular diseases, including stroke, hypertension and arrhythmia, as well as several endocrine disorders [3–7]. A wide range of compounds of RC has been isolated and identified in previous studies; most of which were determined to contain lactones, such as alkylphthalides and phthalide dimmers [8–12]. Ferulic acid (**1**) exhibits a number of interesting biological functions, such as reducing the level of nitrite and oxygen free radicals, lowering blood lipids, resisting bacteria and reducing inflammation [13]. Ferulic acid (**1**) is also used as a biomarker for RC in the Chinese Pharmacopoeia [3]. The lactones found in RC can pass through the blood brain barrier and could therefore be the active ingredients in RC [14]. Senkyunolide I (**2**) can be used to treat migraine [15], whereas senkyunolide H (**3**) can be used to reduce the metamorphose damage of red blood cells (RBC), as well as preventing the aggregation of RBC [16]. Senkyunolide A (**4**) can be used to treat inflammatory processes associated with cerebrovascular diseases [7]. High levels of Z-ligustilide (**5**) can be extremely irritating to the human body and prevent other constituents from achieving their biological efficacies [17, 18]. In addition, levistolide A (**6**) is a dimer of Z-ligustilide (**5**), and therefore directly related to the Z-ligustilide (**5**) content [19].

RC is processed in rice wine prior to being used in clinical applications to enhance its blood circulation properties [20]. RC is generally prepared by stir-frying with rice wine [21]. However, several steamed products are also used in clinical practice [22]. The optimum processing conditions and processing methods should always be explored in detail to ensure that the raw materials reach their maximum efficacy. However, the studies pertaining to the effects of different processing methods on the medicinal properties of RC are scarce, with the majority of these studies focusing specifically on the effects of the processing time [23–25]. It was reported that there are post-harvest variations in the main chemical ingredients of fresh, dried and processed *Ligusticum chuanxiong* [26], but the study compared only the constituents in the final products after processing and there have been no reports pertaining to temporal variations in the constituents during processing. Furthermore, the optimal processing time remains unknown. Processing is dynamic; thus, we should monitor the changes to determine the optimal parameters for processing.

**Fig. 1:**
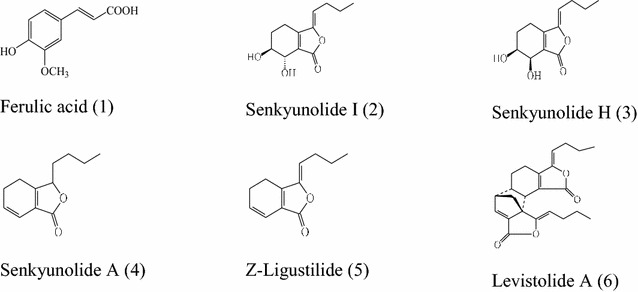
Structures of the six major compounds found in Rhizoma Chuanxiong

Although a wide variety of different chemical constituents are present in RC, ferulic acid, senkyunolide I, senkyunolide H, senkyunolide A, Z-ligustilide and levistolide A are the components commonly used as chemical markers [27] (Fig. 1). This study aimed to investigate the temporal variations of the major constituents of RC by HPLC-DAD-MS during processing to investigate the effects of an adjuvant (e.g., wine), steaming vs stir-frying and the optimal processing time.

## Methods

### Plant materials

The RC, which was produced in Sichuan, was purchased from Qingping Market in Guangzhou, China in 2013. The herbs were authenticated based on their morphological characteristics [4] by Dr. Yi Tao at the School of Chinese Medicine, Hong Kong Baptist University, China. The cleaned RC samples were initially graded according to their sizes, before being macerated in water until they were wet to the core. The RC was then removed from the water and cut into thin slices, before being air-dried at room temperature. The resulting dried pieces of RC consisted of irregular slices of 1–2 mm in thickness, which were divided into four groups. The processing methods and quantities of materials produced in this way are described below in the “Preparation of processed RC” section.

### Reagents and chemicals

Rice wine (Zhejiang Pagoda Band Shaoxing Rice Wine Co., Ltd, China; 15 % alcohol) was purchased from a supermarket in Hong Kong, China. Ferulic acid (**1**) was purchased from the National Institute for the Control of Pharmaceutical and Biological Products (Beijing, China). The chemical standards of senkyunolide I (**2**), senkyunolide H (**3**), senkyunolide A (**4**), Z-ligustilide (**5**) and levistolide A (**6**) used in the current study were isolated from RC in our laboratory [11].

Acetonitrile and formic acid were purchased in liquid chromatography grade from Lab-scan (Bangkok, Thailand). The methanol used for the extraction of the samples was also purchased from Lab-scan. Deionized water was generated using a Milli-Q water purification system (Millipore, Bedford, MA, USA).

### Preparation of processed RC

Four different RC processing methods were employed in the current study. Each processing method was designed according to standard protocols [21]. These four different methods were: (1) stir-frying only, in which the dried RC slices were stir-fried alone in a pan at a temperature of 200 °C to avoid scorching; (2) steaming only, in which the dried RC slices were steamed alone in a steamer; (3) stir-frying with rice wine, in which the dried RC slices (50 g) were soaked in 5 mL of rice wine before frying at 100 °C; and (4) steaming with rice wine, in which the dried RC slices (50 g) were soaked in 5 mL of rice wine before steaming in a steamer. Samples were withdrawn from each process at 5, 10, 15, 20, 25, 30, 35, 40, 50, 55 and 60 min during processing. Sample duplicates were prepared as shown above for analysis.

### HPLC-DAD-MS instrumentation and conditions

An Agilent 1100 high-performance liquid chromatography (HPLC) system equipped with a diode array detector (DAD) and a quadrupole time-of-flight mass spectrometry (TOF–MS) system was used for the qualitative and quantitative analysis of the different samples. The samples were analyzed over an Alltima C18 column (5 µm, 4.6 × 250 mm). Standard solutions of the six chemical standards and RC sample solutions derived from the four different processing methods were prepared and analyzed according to previously reported procedures [11].

## Results and discussion

### Identification of the major components of RC

**Fig. 2:**
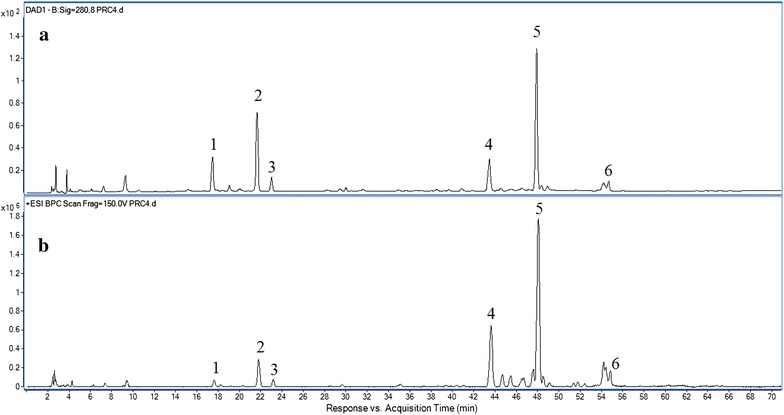
Typical **a** HPLC chromatogram of processed Rhizoma Chuanxiong at 280 nm and **b** TIC chromatogram of this material in the positive ionization mode

**Fig. 3:**
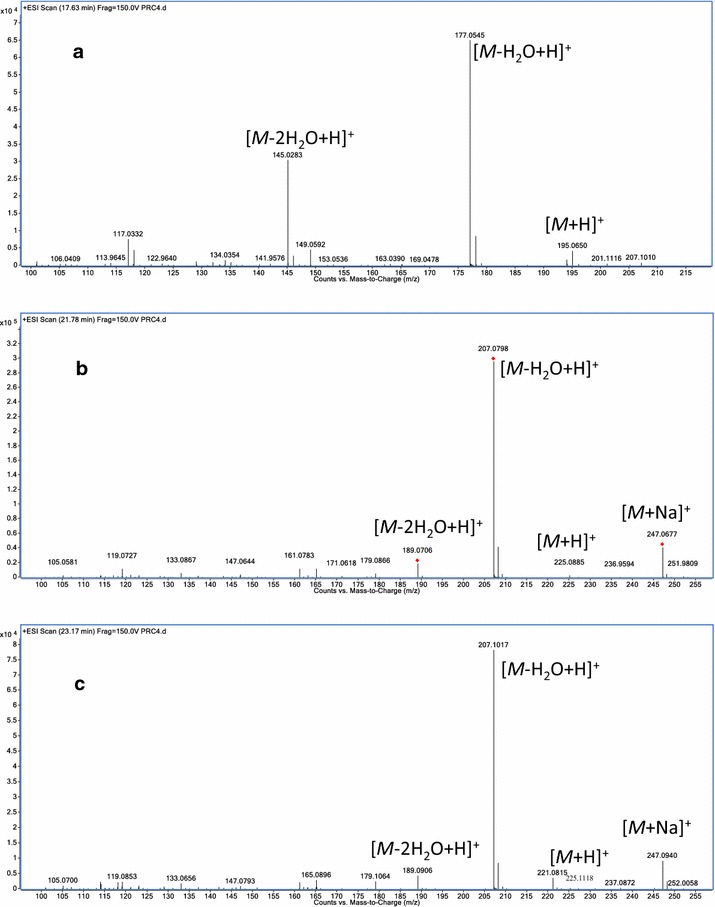
The MS spectra of **a** ferulic acid, **b** senkyunolide I, **c** senkyunolide H

**Fig. 4:**
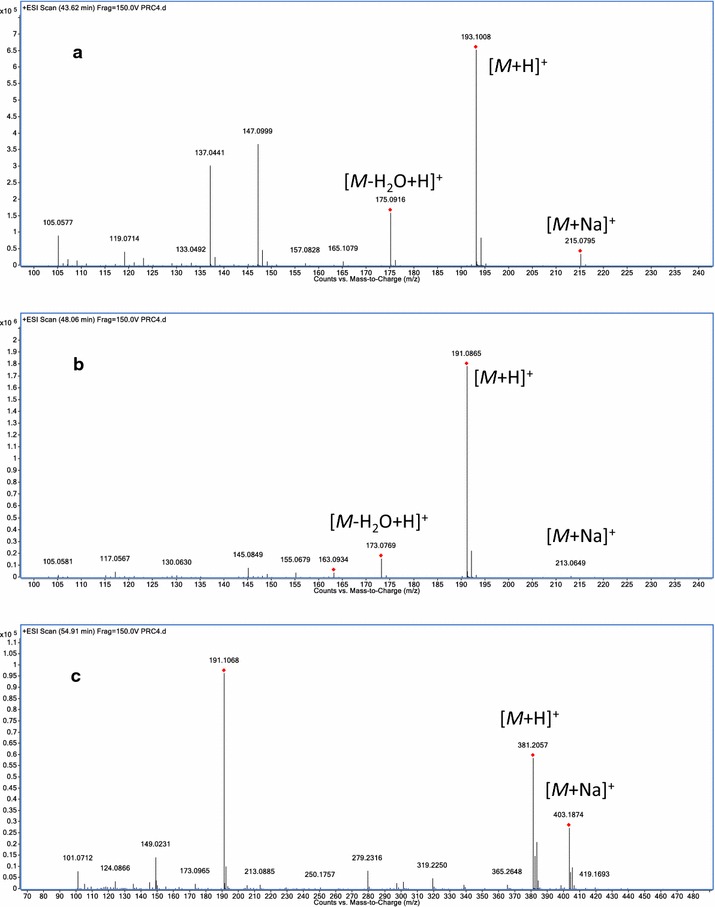
The MS spectra of **a** senkyunolide A, **b** Z-ligustilide and **c** levistolide A in the positive ionization mode

The standard solutions and sample solutions were injected into the HPLC-DAD-MS system described above for analysis. By comparing with standard compounds, six major peaks in the chromatograms of RC were unambiguously identified as ferulic acid (**1**), senkyunolide I (**2**), senkyunolide H (**3**), senkyunolide A (**4**), Z-ligustilide (**5**) and levistolide A (**6**). A typical HPLC chromatogram of RC recorded at 280 nm is shown in Fig. 2 together with a total ion chromatogram (TIC) of this material in the positive ionization mode. The MS spectra of the six different components are shown in Figs. 3 and 4.

### Effect of the processing method on the major constituents

**Fig. 5:**
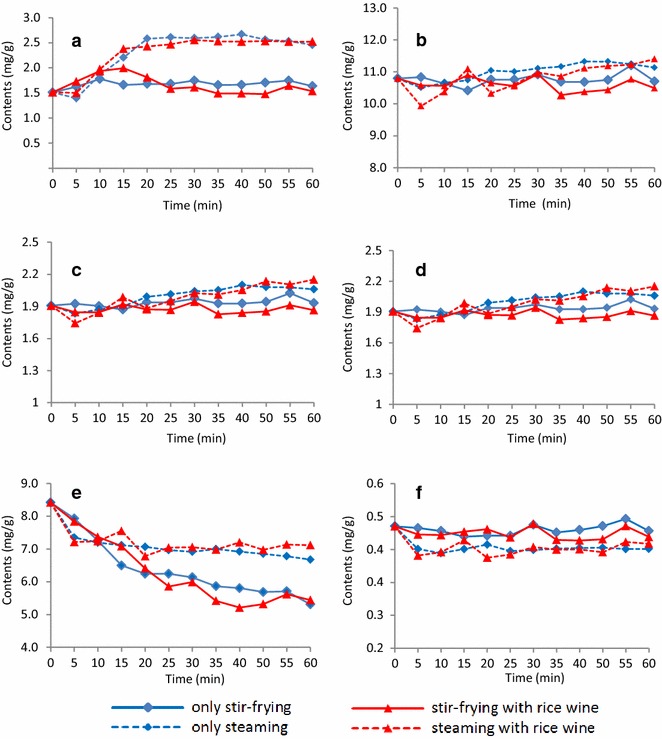
Changes in the **a** ferulic acid, **b** senkyunolide I, **c** senkyunolide H, **d** senkyunolide A, **e** Z-ligustilide and **f** levistolide A contents of the Rhizoma Chuanxiong samples subjected to the four different processing methods over time (*n* = 3)

The RC samples collected from the different processing methods at different time points were analyzed using the present method (Fig. 5). Pronounced variations were observed in the ferulic acid (**1**), senkyunolide I (**2**), senkyunolide H (**3**), senkyunolide A (**4**), Z-ligustilide (**5**) and levistolide A (**6**) contents of the RC samples processed under the different treatment conditions. The ferulic acid (**1**) content of the samples derived from the four different processing methods initially increased significantly until it reached a plateau and remained stable with increasing processing time. The senkyunolide I (**2**), senkyunolide H (**3**) and senkyunolide A (**4**) contents of the samples derived from the four different processing methods also increased slightly with increasing processing time. In contrast, the Z-ligustilide (**5**) content decreased with time in all four cases. Lastly, the levistolide A (**6**) content remained unchanged for the four different processing methods. In general, the amounts of the major constituents were higher after steaming than after stir-frying.

### Optimization of the processing time

**Fig. 6:**
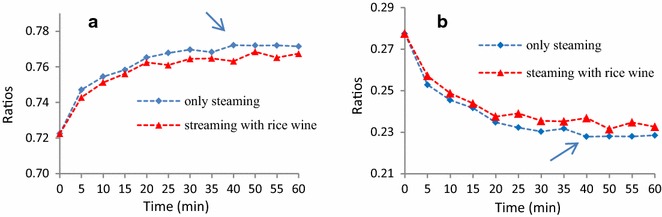
The content ratios of **a** (ferulic acid + senkyunolide I + senkyunolide H + senkyunolide A)/six major compounds; **b** (Z-ligustilide + levistolide A)/six major compounds at different time points for steam-processed Rhizoma Chuanxiong (*n* = 3)

In this study, we considered ferulic acid (**1**), senkyunolide I (**2**), senkyunolide H (**3**) and senkyunolide A (**4**) as active ingredients, whereas Z-ligustilide (**5**) and levistolide A (**6**) were considered to be adverse ingredients. The ratios of the active and adverse ingredients to the six major compounds during the steam processing of RC are clearly shown in Fig. 6. These data were used to determine the optimal processing time of the steam-processing method. Steaming in the absence of an adjuvant, which led to an increase in the ratio of active ingredients as well as a decrease in the ratio of adverse ingredients, was found more suitable for the processing of RC than steaming with rice wine. The curves shown in Fig. 6a initially increased with increasing processing time before reaching a plateau. In contrast, the curves shown in Fig. 6b initially decreased before reaching a plateau. These transitions were observed in a single experiment over a period of 40 min.

## Conclusion

Among the four different methods evaluated for RC processing, steaming was better than stir-frying for preserving high amounts of beneficial chemical markers and affording low amounts of adverse ingredients.

## Abbreviations

RC: Rhizoma *Chuanxiong*
CM: Chinese medicine
HPLC-DAD-MS: high performance liquid chromatography-diode array detector-mass spectrometry
TIC: total ion chromatogram
RBC: red blood cells

## References

[1] Zhao ZZ, Liang ZT, Chan K, Lu GH, Lee LM, Chen HB, Li L (2010). A unique issue in the standardization of Chinese materia medica: processing. Planta Med.

[2] Yi T, Zhu L, Peng WL, He XC, Chen HL, Li J, Yu T, Liang ZT, Zhao ZZ, Chen HB (2015). Comparison of ten major constituents in seven types of processed tea using HPLC-DAD-MS followed by principal component and hierarchical cluster analysis. LWT-Food Sci Technol..

[3] Zhan JY, Zheng KY, Zhu KY, Zhang WL, Bi CW, Chen JP, Du YQ, Dong TX, Lau TW, Tsim KW (2013). Importance of wine-treated *Angelica Sinensis Radix* in Si Wu Tang, a traditional herbal formula for treating women’s ailments. Planta Med.

[4] Committee CP (2015). Chinese pharmacopoeia (2015), part 1.

[5] Ran X, Ma L, Peng C, Zhang H, Qin LP (2011). *Ligusticum chuanxiong* Hort: a review of chemistry and pharmacology. Pharm Biol.

[6] Lim LS, Shen P, Gong YH, Lee LS, Yong EL (2006). Dynamics of progestogenic activity in serum following administration of *Ligusticum chuanxiong*. Life Sci.

[7] Or TCT, Yang CLH, Law AHY, Li JCB, Lau ASY (2011). Isolation and identification of anti-inflammatory constituents from *Ligusticum chuanxiong* and their underlying mechanisms of action on microglia. Neuropharmacology.

[8] Xu X, Ye T, Shuo Y, Yao L, Guang T (2013). Anti-cancer effects of *Ligusticum chuanxiong* Hort alcohol extracts on HS766T cell. Afri J Tradit Complement Altern Med.

[9] Yi T, Kelvin Leung SY, Lu GH, Zhang H, Chan K (2005). Identification and comparative determination of sekyunolide A in traditional Chinese medicine plants *Ligusticum chuanxiong* and *Angelica sinensis* by HPLC with DAD and ESI-MS. Chem Pharm Bull.

[10] Chen XQ, Kong L, Su XY, Fu HJ, Ni JY, Zhao RH, Zou HF (2004). Separation and identification of compounds in Rhizoma chuanxiong by comprehensive two-dimensional liquid chromatography coupled to mass spectrometry. J Chromatogr A.

[11] Yi T, Leung KSY, Lu GH, Chan KC, Zhang H (2006). Simultaneous qualitative and quantitative analyses of the major constituents in the rhizome of *Ligusticum chuanxiong* using HPLC-DAD-MS. Chem Pharm Bull.

[12] Li W, Tang Y, Chen Y, Duan JA (2012). Advances in the chemical analysis and biological activities of chuanxiong. Molecules.

[13] Bourne LC, Rice-Evans C (1998). Bioavailability of ferulic acid. Biochem Bioph Res Co..

[14] Xiong YK, Liang S, Hong YL, Yang XJ, Shen L, Du Y, Feng Y (2013). Preparation of ferulic acid, senkyunolide I and senkyunolide H from *Ligusticum chuanxiong* by preparative HPLC. China J Chin Mater Med..

[15] Wang YH, Liang S, Xu DS, Lin X, He CY, Feng Y, Hong YL (2011). Effect and mechanism of senkyunolide I as an anti-migraine compound from *Ligusticum chuanxiong*. J Pharm Pharmacol.

[16] Hong M, Dong ZB, Zhu Q (2003). Effects of ferulic acid, senkyunolide H and senkyunolide I on erythrocytes. Lishizhen Med Mater Med Res.

[17] Zheng YZ, Choi RC, Li J, Xie HQ, Cheung AW, Duan R, Guo AJ, Zhu JT, Chen VP, Bi CW, Zhu Y, Lau DD, Dong TT, Lau BW, Tsim KW (2009). Ligustilide suppresses the biological properties of Danggui Buxue Tang: a Chinese herbal decoction composed of Radix Astragali and Radix Angelica Sinensis. Planta Med.

[18] Zhan JY, Zheng KY, Zhu KY, Bi CW, Zhang WL, Du CY, Fu Q, Dong TT, Choi RC, Tsim KW, Lau DT (2011). Chemical and biological assessment of Angelicae Sinensis Radix after processing with wine: an orthogonal array design to reveal the optimized conditions. J Agr Food Chem..

[19] Yan RL, Guo L, Pan MF, Zhou XL (2012). Determination of ligustilide and levistolide A in *Ligusticum chuanxiong* Hort by HPLC. J Anhui Agr Sci..

[20] Huang QW, Huang YL, Han L, Xie XQ (2007). Pharmacological equivalent test in Chuanxiong formula granule. West China J Pharm Sci..

[21] People’s Republic of China Ministry of Health, Bureau of Pharmaceutical Affairs. Processing Standards of the People’s Republic of China. Beijing: People’s Health Press; 1988. p. 14.

[22] Zhang DJ, Zhao ZZ, Chen HB (2011). Comparative study on decoction pieces of traditional Chinese crude drugs from Hong Kong and mainland of China. Hong Kong Chin Med J.

[23] Li BP, Feng QR, Ou BX, He GL (2012). Optimization of microwave processing technology of wine-processed Rhizoma Chuanxiong with orthogonal design. Tradit Chin Drug Res Clin Pharm..

[24] Xie ZD, Yi DY, Fang YQ, Guo JS (2012). Studies on traditional pharmaceutical processing for chuanxiong Rhizome in medical history and modern research. Chin J Exp Tradit Med Formulae..

[25] Fang JY, Zhu L, Yi T, Zhang JY, Yi L, Liang ZT, Xia L, Feng JF, Xu J, Tang YN, Zhao ZZ, Chen HB (2015). Fingerprint analysis of processed Rhizoma Chuanxiong by high-performance liquid chromatography coupled with diode array detection. Chin Med..

[26] Li SL, Yan R, Tam YK, Lin G (2007). Post-harvest alteration of the main chemical ingredients in *Ligusticum chuanxiong* HORT (Rhizoma Chuanxiong). Chem Pharm Bull.

[27] Zhang YN, Yue XF, Zhang ZQ (2004). Study on the interactions between four components in *Ligusticum chuanxiong* rhizome and acceptors on cardiac muscle membrane. China J Chin Mater Med..

